# Effects of parental age and polymer composition on short tandem repeat de novo mutation rates

**DOI:** 10.1093/genetics/iyae013

**Published:** 2024-01-31

**Authors:** Michael E Goldberg, Michelle D Noyes, Evan E Eichler, Aaron R Quinlan, Kelley Harris

**Affiliations:** Department of Genome Sciences, University of Washington, Seattle, WA 98195, USA; Departments of Human Genetics and Biomedical Informatics, University of Utah, Salt Lake City, UT 84112, USA; Department of Genome Sciences, University of Washington, Seattle, WA 98195, USA; Department of Genome Sciences, University of Washington, Seattle, WA 98195, USA; Howard Hughes Medical Institute, University of Washington, Seattle, WA 98195, USA; Departments of Human Genetics and Biomedical Informatics, University of Utah, Salt Lake City, UT 84112, USA; Department of Genome Sciences, University of Washington, Seattle, WA 98195, USA; Computational Biology Division, Fred Hutchinson Cancer Research Center, Seattle, WA 98109, USA

**Keywords:** mutation rate, tandem repeats, DNA replication, human evolution, molecular evolution

## Abstract

Short tandem repeats (STRs) are hotspots of genomic variability in the human germline because of their high mutation rates, which have long been attributed largely to polymerase slippage during DNA replication. This model suggests that STR mutation rates should scale linearly with a father's age, as progenitor cells continually divide after puberty. In contrast, it suggests that STR mutation rates should not scale with a mother's age at her child's conception, since oocytes spend a mother's reproductive years arrested in meiosis II and undergo a fixed number of cell divisions that are independent of the age at ovulation. Yet, mirroring recent findings, we find that STR mutation rates covary with paternal and maternal age, implying that some STR mutations are caused by DNA damage in quiescent cells rather than polymerase slippage in replicating progenitor cells. These results echo the recent finding that DNA damage in oocytes is a significant source of de novo single nucleotide variants and corroborate evidence of STR expansion in postmitotic cells. However, we find that the maternal age effect is not confined to known hotspots of oocyte mutagenesis, nor are postzygotic mutations likely to contribute significantly. STR nucleotide composition demonstrates divergent effects on de novo mutation (DNM) rates between sexes. Unlike the paternal lineage, maternally derived DNMs at A/T STRs display a significantly greater association with maternal age than DNMs at G/C-containing STRs. These observations may suggest the mechanism and developmental timing of certain STR mutations and contradict prior attribution of replication slippage as the primary mechanism of STR mutagenesis.

## Introduction

Short tandem repeats (STRs) are genomic elements composed of repeating nucleotide motifs typically of length 1–6 bp (Weber and Wong 1993). Sometimes also known as microsatellites and minisatellites, they mutate at rates orders of magnitude greater than nonrepetitive loci, a feature that results in high levels of diversity that enabled studies of genetic diversity in the pregenomic era (Weber and Wong 1993; Spencer *et al*. 2000). Despite this classical importance, STRs have fallen behind relative to single nucleotide variants (SNVs) in terms of our understanding of how germline mutation rates vary in humans, what environmental or genetic conditions may contribute to this variance, and how their variation broadly shapes complex traits and genomic evolution.

STRs are numerous—they encompass nearly 3% of the human genome, a similar proportion to that of coding regions (*Lander et al. 2001*). Until recently, our understanding of how specific STR loci affect traits remained limited to a small number of large effect size loci, though the molecular underpinnings of their associations still remain largely unclear (McGinty and Sunyaev 2023). Germline and somatic expansion at these loci have long been implicated in Mendelian developmental and neurodegenerative disorders (Sherman *et al*. 1985; Gatchel and Zoghbi 2005; Mitra *et al*. 2021; Nurk *et al*. 2022). More broadly, variation genome wide has recently demonstrated association with complex traits and gene expression (Gymrek *et al*. 2016; Fotsing *et al*. 2019; Margoliash *et al*. 2022). Accordingly, some STRs are inferred to be under purifying selection, regardless of their instability (Gymrek *et al*. 2017; Mitra *et al*. 2021). Children diagnosed with simplex (likely noninherited) autism spectrum disorder (ASD) have been found to harbor de novo mutations (DNMs) at STRs inferred to be more deleterious than those identified in their unaffected siblings (Mitra *et al*. 2021). These recent findings suggest that STRs’ complex genotype–phenotype interactions and evolutionary history may have similar complexity to those attributed to SNVs and structural variation.

Both SNV and STR mutation rates depend strongly on paternal age (Kong *et al*. 2012; Forster *et al*. 2015). This dependency was classically explained by the fact that male germ cells replicate throughout the reproductive lifespan, assuming that most mutations originate as errors in replication (Goodman 1985; Drost and Lee 1995). In the case of SNVs, recent work has cast doubt on this model and has begun to support DNA damage as a substantial source of SNVs (Goodman 1985; Drost and Lee 1995; Gao *et al*. 2019; Wu *et al*. 2020). The proportion of SNVs inherited from the paternal lineage, known as *ɑ*, remains strikingly stable with parental age, which should not be the case if most mutations are replicative in origin and the ratio of paternal to maternal cell divisions increases with age (Gao *et al*. 2019). Although the female germline does not continue to replicate after birth, the ratio of male to female germline mutations appears to remain largely constant regardless of developmental stage. Nevertheless, analyzing how the rates of different types of mutations covary with parental age and sex can provide clues to mutational mechanisms that may be more associated with replication errors or with DNA damage.

Although we have less information on how life history impacts STR mutagenesis, the high mutation rate at these loci has long been attributed primarily to polymerase slippage during S phase replication (Klintschar *et al*. 2004; Forster *et al*. 2015). Replication slippage typically causes STR expansions or deletions by one or more repeat units (Brinkmann *et al*. 1998; Sun *et al*. 2012; Amos *et al*. 2015; Mitra *et al*. 2021). Historically, studies have found no genome-wide association of STR mutation rates with maternal age (maternal age effect) but have found a strong association with paternal age (paternal age effect) (Sun *et al*. 2012; Forster *et al*. 2015). At face value, these age effects seemed to bolster the theory that STRs mutate only during DNA replication in S phase. Given the lack of maternal germline replication after the future mother's birth, we would expect replication-driven mutations to exhibit a paternal age effect but no maternal age effect (Drost and Lee 1995). However, previous studies of STR parental age associations draw upon much less data than comparable studies of single nucleotide mutation rates. Certain STR loci have been shown to accumulate mutations in postmitotic cells, indicating that damage could play some role in STR mutagenesis independent of cell divisions (Gonitel *et al*. 2008). For example, somatic expansion in postmitotic striatal medium spiny neurons of the CAG/CAA repeat that encodes a polyglutamine tract in the *huntingtin* gene causes Huntington's disease, a highly heritable and penetrant neurodegenerative disease with adolescent/early adulthood onset (Lee *et al*. 2019; Wright *et al*. 2019). Furthermore, in vitro studies have shown evidence that the rate of expansions and deletions at STRs may not be fully explainable by strand slippage alone (Ananda *et al*. 2011).

Although directly proving a link between a mutational pattern and its causal mutational pathway can be challenging, it is possible to infer potential molecular mechanism by examining the covariance of mutation rate with polymer complexity and nucleotide content. Local mutation rate, chromatin state, and many other genomic features covary with nucleotide content (Hwang and Green 2004; Alexandrov *et al*. 2013, 2020; Goldberg and Harris 2022). This broad phenomenon can be attributed to the fact that nucleotide content is highly predictive of local DNA conformation, or 3-dimensional molecular shape, which in turn associates with the types of molecules likely to interact with a locus and the probability of those interactions (Rohs *et al*. 2009; Lazarovici *et al*. 2013; Abe *et al*. 2015; Liu and Samee 2023). Similarly, certain types of STRs are known to form unique structures different from the canonical double helix of B DNA; for example, some guanine-rich repeats form fragile cruciforms (McGinty and Sunyaev 2023). Different tumor types display variance in mutational spectra and signatures in mutations at repetitive DNA elements, indicating tissue- or tumor-specific mutation rate modifiers (Alexandrov *et al*. 2020). When applied to polymorphic and de novo germline SNVs, these types of analyses have revealed significant diversity in the spectra of mutations accumulating in different populations, families, or species (Hwang and Green 2004; Harris 2015; Harris and Pritchard 2017; Carlson *et al*. 2020; Bergeron *et al*. 2023). Similar approaches have helped identify variance in mutational pathways active at STRs: recently, a small set of families were discovered to harbor a mutator allele that affects indels at TNT repeats (Reijns *et al*. 2022; Maksimov *et al*. 2023).

In this study, we sought to further describe how STR mutation rates vary as a function of parental age and sex to better understand the developmental timeline and possible mutational mechanisms. To do so, we analyzed a data set of DNMs at STR loci found in 1593 families comprising two parents and two children (quads), one of whom has been diagnosed with a simplex case of ASD without prior family history (Fischbach and Lord 2010). These families were recruited, in part, to study how rare DNMs contribute to ASD as a subset of the Simons simplex collection (SSC) (Fischbach and Lord 2010). Our work aims to clarify how parental age and sex impact both autism risk and deleterious DNM load, as we further characterize how these variables effect the distribution of fitness effects (DFE) of STR DNMs. Very recently, a study examining nearly 77k STR DNMs in over 6,000 trios identified a significant association of DNM rate with maternal age, dispelling a simplistic model of STR mutagenesis in the germline (Kristmundsdottir *et al*. 2023). We confirm the presence of this maternal age effect in an independent data set and demonstrate its robustness to noise typical of STRs genotyped with short-read sequencing. Furthermore, we examine how parental age effects are modified by certain genomic features, identifying specific DNA motifs enriched for the maternal age signal.

## Materials and methods

### DNM filtering

We analyzed published STR genotypes and DNMs in the SSC as previously described in Mitra *et al*. (2021). However, we implemented several additional filtering strategies to account for possible cryptic sources of bias in the data set. In particular, we noticed that DNMs at loci where one parent is homozygous and the other is heterozygous are more likely to originate on the chromosome inherited from the homozygous parent rather than the heterozygous parent (Supplementary Fig. 1). We hypothesized that this observation might be the result of allelic dropout events: if a parent is heterozygous at a locus but incorrectly genotyped such that they appear homozygous, their child could inherit an allele that is falsely identified as a de novo event. Similar strategies to account for possible allelic dropout in this data set have been previously described; we developed a new, strict, read-based approach to accounting for this possible artifact (Mitra *et al*. 2021).

Using a read-based approach, we filtered a starting set of 175,290 de novo STR calls identified by MonSTR. We used pysam to assess the number of Illumina reads with the de novo allele in a child and its parents. Importantly, we did not perform a local realignment step like Mitra *et al.* but instead opted to use the original alignments to GRCh38. First, we selected any primary reads aligned to the starting coordinate of the STR with a mapping quality of >60. We retained only reads that not only spanned the length of the longest observed STR allele in the family but also included 10 bp of flanking sequence on either end of the STR, excluding soft-clipped bases. For every read that completely spanned the length of the longest STR sequence and its flanking bases, we queried the sequence at those coordinates. To determine the length of the STR allele in a given read, we simply traversed the sequence until we found the first instance of the STR motif and then counted the number of continuous STR motifs that followed. Once we reached the end of the STR, we checked the 10 bases preceding the start and following the end to ensure that they matched the reference sequence that flanks the STR. Reads with interruptions in the STR sequence or more than one mismatch between the observed and reference flanking were discarded. If there were no reads with the de novo allele in the child, we called the variants a false positive. If there were one or more reads with the de novo allele in either parent, we called the variant inherited. All de novo variants with no read support in either parent and at least one supporting allele in the child were labeled true events, for a final de novo STR callset of 56,925 variants.

We generated long-read sequencing data using Pacific Biosciences high fidelity (HiFi) and Oxford Nanopore Technologies (ONT) for two families from our data set in order to further validate de novo variants and assess our validation strategy (a total of 157 STR variants across four children) (Supplementary Table 1). For the parents and child, we aligned HiFi and ONT data to GRCh38 and then manually examined the length of the STR allele in each aligned read with a mapping quality of >60. Using the same criteria (at least one read with the de novo allele in the child and no reads with the de novo allele in the parents), we determined whether each variant was a true de novo event. Upon visual inspection, we found that only 52.5% (31/59) of nonhomopolymer DNMs have no evidence of being inherited (24.8% of all DNMs [39/157]). Furthermore, we found that only 8% of putative STR DNMs at homopolymer loci showed no evidence of being inherited from parents or falsely genotyped in the child. This observation further motivated our decision to exclude all homopolymer DNMs from the analysis. Based on the long-read data, we predict our filter has an accuracy of 92.3%, sensitivity of 94.8%, and specificity of 91.5%. Furthermore, the bias toward DNM phasing to homozygous parents we observed with the full data set was attenuated when applying these filters (Supplementary Fig. 1).

The sensitivity of this filtration strategy, however, may be lower at some loci and alleles. Each read mapping to an STR locus supports a certain allele length, here measured in a number of repeat units; GangSTR uses the amount of read support for allele lengths to call a genotype. However, at some loci, reads support a variety of possible allele lengths. For example, at a site where parents have genotypes [10, 10] and [10, 10] but frequently we observe reads supporting an allele of length [11], we would erroneously filter possible de novo [11] alleles in a child. We were therefore motivated to further assess the variance in sensitivity of our filter, i.e. how frequently would we erroneously filter out a putative STR DNM. Given a family with parental genotypes [10, 10] and [10, 10] and a proband genotype of [10, 11] (putative de novo allele [11]), we searched for any other set of parents with the exact same genotypes (sex nonspecific) and no children with the same putative de novo allele; these are “positive” couples, from whom a putative [11] allele was unlikely to have dropped out from genotyping. In these positive couples, we counted the number of reads that supported the target family's putative de novo allele to assess the frequency with which we would erroneously flag a couple for possible allelic dropout. We found that sensitivity differed greatly between loci and sometimes between alleles at the same locus (Supplementary Fig. 2). We could not reliably predict which loci and genotypes had high false negative rates; calculating the empirical false negative rate was only possible for loci and genotypes common among the SSC parents. Implementing an updated filtration strategy allowing >0 reads supporting the putative de novo allele at certain loci could bias us against rare genotypes, especially those uncommon in families of predominantly European ancestry, which comprise the bulk of the SSC (Wilfert *et al*. 2021). Thus, we chose to maintain our strict filtering technique and accounted for possible variance in sensitivity by calculating and regressing with an accurate denominator, as described in the following section.

### Denominator calculation

At sites with high sequencing noise or parental diversity in alleles, only a subset of possible DNMs can be confidently called. In the following descriptions, alleles are annotated by their number of repeat units. For example, at a site where the parents have genotypes [10, 10] and [11, 11], a 10 -> 11 or an 11 -> 10 DNM resulting in either a [10, 10] or a [11, 11] genotype in the child would be indistinguishable from (and perhaps more parsimoniously explained by) allelic dropout in the child. Furthermore, if ≥1 reads from the [11, 11] parent indicated a 12 allele, observing the 12 allele in the child could again indicate dropout over mutation. However, any DNM could be discoverable at a site where both parents are [10, 10] with no reads mapping any other alleles. These concerns affect the denominator for each trio, i.e. how many sites are we able to discover DNMs. To account for variance in the denominator as a possible artifactual source of the association of STR DNM rate with maternal age, we applied an extra set of filters requiring 100% discoverability for all DNMs observed in a trio. For a given mutation size of *i* repeat units, a site is fully discoverable if and only if for every parental allele *a* in all parental alleles *A*, no reads in the parents map to any allele of size *a* ± *i*. Note that this filter excludes possible DNMs of size *i* that occur at sites where not all DNMs of size *i* are discoverable. The resulting mutation rate is calculated as


$$\begin{array}{rl} & \mathrm{mutation}\;\mathrm{rate}\;=\\ & \;\;\;\;\underset{i}{\sum }\frac{\#\;\mathrm{mutations}\;\mathrm{of}\;\mathrm{size}\;i}{\#\;\mathrm{total}\;\mathrm{sites}\;\mathrm{discoverable}\;\mathrm{for}\;\mathrm{mutations}\;\mathrm{of}\;\mathrm{size}\;i}. \end{array}$$

We calculated the mutation rate across *i* in [1, 2, 3], which accounted for 98% of the DNMs, which passed filters described in the previous section.

To implement this filter, we used the same underlying framework of our filtering script to identify STR sites where we could observe a DNM of a given number of motifs. For each pair of parents in the dataset, we examined every STR site that was genotyped in all four parental haplotypes in Illumina data aligned to GRCh38. Borrowing code from our STR filtering script, we selected passing reads and estimated the length of the STR in each read. We classified every observed allele as an “uncallable mutation”—if an allele was observed in even one parental read, it would never pass our de novo filtering script. Unfortunately, this filter left us with only 833 maternally derived mutations, which gave us insufficient power to observe a significant maternal age effect on their rate per child (*P* = 0.12, generalized linear model [GLM]). These validated mutations are more challenging to phase with our methods, given the high proportion from sites with no diversity in parental alleles (31% vs 15% of DNMs that passed filters in prior section). However, a regression of the mutation rate of all mutations regardless of phase and against both maternal and paternal ages found that both parental ages were significantly positively associated with the rate (*P*= 1 ∗ 10^−11^ and 0.012 for paternal and maternal ages). This model was significantly better than one that did not include maternal age as a covariate (delta Akaike information criterion [AIC] = −4). Furthermore, Poisson GLMs regressing the number of fully discoverable mutations (the numerator in the above equation) against parental age including an offset of the sum of the number of loci discoverable for mutations of sizes 1, 2, and 3 (the denominator in the above equation) found significant paternal and maternal age effects, respectively (*P* < 2.2 ∗ 10^−16^ and *P* = 4.41 ∗ 10^−9^ for paternal and maternal age effects, respectively, log link). This indicated to us that, although the maternal age effect appeared robust to differences in denominator, this approach could result in a lack of statistical power for any further analyses. Therefore, in the main figures, we continued to use the filtering approach described in the previous section that did not account for the discoverability of sites.

### Determining power to detect the postzygotic effect of maternal age

We find no evidence that the maternal age effect on STR DNMs is due to a heightened amount of postzygotic mutagenesis in the child's early development, in contrast to what has been detected in SNV DNMs (Gao *et al*. 2019). However, due to the low number of nonhomopolymer STR DNMs that pass our filters and can be phased to a parental lineage, we may be limited in our power to detect small amounts of this postzygotic mutagenesis. To determine our power, we simulated paternally derived mutations for children in the SSC at a variety of postzygotic maternal age effects and tested for correlation between maternal age and paternally derived DNMs while conditioning on paternal age, as in Supplementary Fig. 6. If some fraction *f_z_* of the additional mutations associated with maternal age occur after zygote formation, we assume that these mutations will be randomly distributed between the paternally and maternally inherited chromosomes. Thus, given paternal and maternal ages *age*_pat_ and *age*_mat_ per child, paternal age *y*-intercept and slope *β*_0,*p*_ and *β*_1,*p*_, and maternal age slope *β*_1,*m*_, we simulated paternally derived STR DNMs as a Poisson distributed random variable given by *λ*:


$$\mathrm{Pois}(\lambda =\beta_{0,p}+\beta_{1,p}\;*\;age_{p}+\beta_{1,m}\;*\;f_{z}\;*\;age_{m})$$

**Fig. 1.**
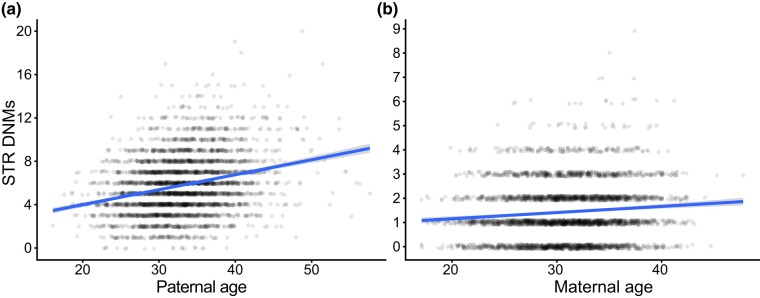
Significant maternal and paternal age effects on STR DNM rate. a, b) Number of paternal and maternal nonhomopolymer STR DNMs per offspring in the SSC are plotted and regressed against paternal and maternal age at birth, respectively. *Y*-axis jitter is added to points for readability. The regression line is a Poisson GLM fit with an identity link function in ggplot2.

Beta coefficients were taken from the Poisson linear models in Fig. 1. Poisson sampling was done in R 4.1.1 using function rpois(). For each round of simulation, we drew paternally derived STR DNMs for each child, grouped children into nonoverlapping pairs of identical rounded paternal ages, and calculated the difference in maternal ages and the difference in simulated paternally derived STR DNMs between the child born to younger and the child born to older parents. We then estimated the significance of the positive correlation between the maternal age difference and paternal STR DNM difference for all pairs of children with a one-sided Spearman's rank correlation coefficient and saved the *P*-value. For values of *f_z_* < 0.4, we appear to have no power to detect significant postzygotic effects; our power is still limited even for stronger postzygotic effects. More efficient phasing methods, cleaner genotyping, and a larger set of families would all increase statistical power for future iterations of this analysis.

### Regression framework

To model the effects of parental age on the number of STR DNMs, we typically employed Poisson regressions with identity link function in R (version 4.1.1), following prior work (Jónsson *et al*. 2017; Sasani *et al*. 2019). The identity link specifies a linear relationship between the parental age and the expected number of mutations in a given child at that age. For example, we used the following model to estimate the maternal age effect on a number of maternally derived nonhomopolymer STR DNMs.

glm(maternal_nonhomopolymer ∼ motherAgeAtBirth, … family = poisson(link = “identity”)).

**Fig. 2.**
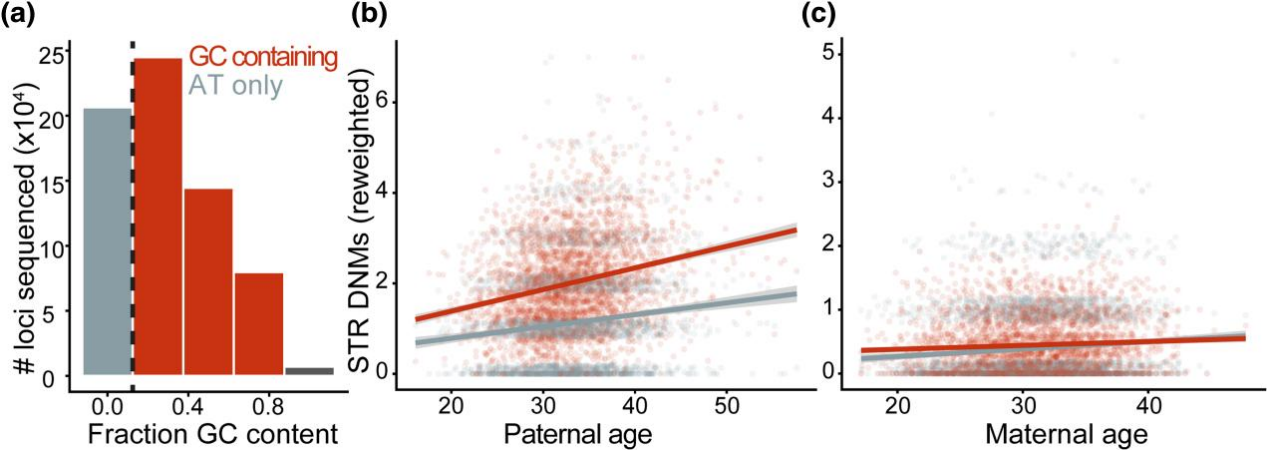
Nucleotide content-specific effects of maternal age. a) We split loci into two bins as a function of their nucleotide content: AT-only and GC-containing. b, c) Association of paternal and maternal age with STR DNM rate as a function of nucleotide content. In each scatterplot, each trio in the SSC is represented twice. The number of STR DNMs phased to the respective parental lineage is plotted as a function of the respective parental age for AT-only and GC-containing STRs. For readability and to account for differences in panel size, the number of GC-containing STR DNMs is rescaled by the ratio of AT-only to GC-containing loci in the panel. We account for panel size differences in the models described in the main text with an offset covariate, though lines plotted here are Poisson GLMs with identity link functions fit to rescaled mutation counts with ggplot2. Coefficients are significant beyond *P* = 0.05.

We similarly modeled the effects of nucleotide content and parental age as a Poisson GLM while allowing linear interaction between parental age nucleotide content of STRs (Fig. 2). To account for differences in the number of AT-only and GC-containing nonhomopolymers in our panel, we included an offset term that directly accounts for differences in denominators for rates, though are more straightforward to implement with a log rather than an identity link function. For example, we used the following model to estimate how nucleotide content modified the maternal age effect:

glm(n_muts ∼ motherAgeAtBirth + gc_content + offset(log(denom)…, family = poisson(link = “log”)).

In the above model, gc_content is a binary variable classifying a locus as being AT-only or GC-containing; denom is the number of loci in the panel matching the gc_content classification.

### Comparison to Sun *et al*. (2012)

Prior research has detected no significant maternal age effect on the rate of maternally derived STR DNMs (Sun *et al*. 2012). We hypothesized that this study was underpowered to discover the maternal age effect we observed. To test this hypothesis, we subset our loci roughly to the ones that were included in the prior study. Sun *et al*. examined 2,477 loci of a subset of 5,136 loci originally identified to estimate a human genetic map (Kong *et al*. 2002; Sun *et al*. 2012). These loci are all (AC)*_n_*/(TG)*_n_* dinucleotide repeats; their discovery process is described in several earlier publications (Weissenbach *et al*. 1992; Dib *et al*. 1996). The list of 2,477 loci is not available, so we analyzed the superset of 5,136 loci. We found the ∼100- to 400-bp genomic region in GRCh38 associated with the original marker using tables from the UCSC Table Browser. 2040 AC (CA, GT, or TG) repeats in our panel that fell within these windows were included in our analysis. GLMs regressing paternal or maternal mutation rate against paternal or maternal age, respectively, found a significant paternal but no significant maternal age effect (*P* = 2.81 ∗ 10^−7^, 0.165, respectively). A possible explanation for the lack of significant maternal age effect could be the lower number of transmissions observed at AC dinucleotides in Sun *et al*. While their study sequenced 24,832 trios at 2,477 loci for a total of 6.15 ∗ 10^7^ transmissions at AC dinucleotides, our study examined 3,172 at 55,830 AC dinucleotide loci alone for 1.77 ∗ 10^8^ transmissions (and 6.83 ∗ 10^5^ nonhomopolymer STR loci for 2.17 ∗ 10^9^ total transmissions).

## Results

We examined STR DNMs in a cohort of 1,593 quad families recruited as a part of the SSC based on the diagnosis of a simplex case of ASD in a single child (Fischbach and Lord 2010). These probands have been shown to harbor an enrichment of both SNV and STR DNMs that may affect neurodevelopment and lead to ASD (Turner *et al*. 2017; Mitra *et al*. 2021). We used mutation calls from Mitra *et al*. 2021. As described previously, the families were sequenced to 35× coverage, and diploid genotypes at STR loci were inferred using GangSTR (Fischbach and Lord 2010; Mitra *et al*. 2021). This database of STR DNMs is the largest compiled to date. DNMs were called using MonSTR, a likelihood-based caller with an accuracy of 90%; this accuracy was estimated based on validation with capillary electrophoresis (Mitra *et al*. 2021). Stringent filters were placed on the genotype calls as described in Mitra *et al*. 2021. We developed further stringent filters on the DNM calls to account for allelic dropout in parents at high diversity sites that result in false positive DNM calls in the children; these filters were further validated with Pacific Biosciences HiFi and ONT long-read sequencing generated for two quads (*Materials and methods*) (Supplementary Figs. 1 and 2; Supplementary Table 1). Briefly, these filters involve removing any DNM at a locus at which one or more reads observed in the parents matches the putative de novo allele observed in the child and resulted in filtering out 43% of nonhomopolymer DNMs (30,623 out of 71,581). Similarly to Mitra *et al*., we excluded homopolymers from all analyses unless otherwise specified, as homopolymers are challenging to genotype with short-read next-generation sequencing methods (Stoler and Nekrutenko 2021). As fully described in Mitra *et al*., mutations were phased to their parental lineages based solely on the observed alleles in parents and child without explicitly recreating the surrounding haplotype. If the inherited allele in a child could be assigned unambiguously to 1 parent's lineage, the de novo allele was phased to the other parent's lineage (Mitra *et al*. 2021). We were able to phase 56% of the nonhomopolymer DNMs that passed our filters. Following these filtering steps, the mean number of STR DNMs per child was 12.91; the mean number of paternally and maternally phased DNMs was 5.80 and 1.43, respectively (95% confidence intervals [CIs] 12.76–13.06, 5.70–5.89, and 1.39–1.49, respectively). Affected probands and their unaffected siblings respectively harbored 13.11 and 12.72 STR DNMs (95% CI 12.96–13.25 and 12.57–12.87, respectively).

We separately regressed the number of maternally and paternally phased mutations per child against their mother's and father's ages at their birth, respectively, using Poisson regressions with identity link functions (this regression model was used throughout the study unless otherwise specified). Our analysis confirms the strong paternal age effect on the rate of paternally phased STR mutations that has been reported in this and other STR DNM data sets (Fig. 1a) (0.14 paternal mutations/year, SE = 8.01 ∗ 10^−3^, *P* = 1.11 ∗ 10^−66^). In line with other recent findings, we also observe a significant effect of maternal age on maternally inherited STR DNMs (Fig. 1b) (0.025 maternal mutations/year, SE = 4.42 ∗ 10^−3^, *P* = 8.11 ∗ 10^−9^) (Kristmundsdottir *et al*. 2023). The maternal age model does not demonstrate significant overdispersion, which can lead to artificially deflated *P*-values (*P* = 0.27, dispersion = 1.02, overdispersion test). Maternal age remains a significant predictor of the maternal STR mutation rate even when we include paternal age as a covariate, indicating that phasing errors are not likely to explain the signal (*P* = 4.20 ∗ 10^−6^). An exponential model of the maternal age effect fits slightly better than the linear model (delta AIC = −0.2), whereas the linear model is the superior fit for the paternal age effect (delta AIC = −5). This finding mirrors the observation of an exponential maternal age effect on the SNV rate presented in Gao *et al*. (2019). Interestingly, neither paternal nor maternal age was significantly associated with the DNM rate at homopolymers (*P* = 0.062 and 0.816 for paternal and maternal age effects, respectively) (Supplementary Fig. 3). Although this observation could support an association between parental age effects and variance in a mutational pathway that does not affect homopolymers, it may also reflect the difficulty of accurately genotyping homopolymers and that any parental age effects are overwhelmed by the noise of sequencing errors (Stoler and Nekrutenko 2021). We replicated these analyses accounting for possible additional bias in genotyping and still observed a significant association between maternal age and DNM rate (*Materials and methods*).

Prior to Kristmundsdottir *et al*. (2023), studies found no significant maternal age effect on the STR DNM rate, but most have used several orders of magnitude fewer loci and DNMs (Sun *et al*. 2012; Forster *et al*. 2015). To determine if these prior studies were simply underpowered to discover the maternal age effect, we repeated our regression-based inference of the DNM rate after restricting to the subset of loci analyzed in a prior study by Sun *et al*. (2012) that found no maternal age effect. Although this study included more trios than ours, they used a far smaller number of loci—a subset of 2,477 STR sites from a larger set of ∼5,000 (AC)*_n_*/(TG)*_n_* dinucleotide repeats used as markers to infer recombination maps (Kong *et al*. 2002). Using the superset of Kong *et al*. sites that mapped to loci in our panel (*n* = 2,040), we found significant association of the rate of paternally derived DNMs but no such association of maternally derived DNMs with maternal age (*P* = 2.81 ∗ 10^−7^ and 0.165 for paternal and maternal age effects, respectively; linear regression) (Supplementary Fig. 4) (*Materials and methods*). These results indicated that a low number of loci in prior studies have resulted in insignificant statistical power to discover a maternal age effect.

Recent studies of de novo SNVs have found that the fraction of phased mutations arising from the paternal lineage, known as *ɑ*, does not depend on parental age; instead, the father contributes about 3/4 of mutations regardless of the total mutation load or the ages of the parents (Gao *et al*. 2019). In contrast to this, we find a significant positive association between *ɑ* and paternal age, indicating that the paternal STR mutation rate increases disproportionately to the maternal mutation rate (0.0023 increase in paternal fraction per year, *P* = 4.99 ∗ 10^−3^, quasi-binomial GLM with identity link function) (Supplementary Fig. 5). This observation likely supports replication as the dominant mechanism of STR DNMs, though the weak slope and nonzero maternal age effect indicate that damage independent of replication still contributes.

Part of the maternal age effect on single nucleotide substitutions has been attributed to a higher rate of postzygotic mutagenesis in older mothers (Gao *et al*. 2019). To examine whether the same might be true for STRs, we tested for covariance between maternal age and the mutation rate of STRs occurring on paternally inherited chromosomes, controlling for variance in paternal age (Gao *et al*. 2019). After controlling for paternal age, any correlation of mutation rate on paternally inherited chromosomes with maternal age could indicate that older mothers harbor a higher postzygotic mutation rate. To do this, we randomly sampled nonoverlapping pairs of children born to fathers of the same age (±6 months). For each pair of children, we calculated the differences in maternal ages and the additional number of STR DNMs phased to the father identified in the child born to the older mother. We detected no significant correlation between maternal age and paternally phased STR DNMs, conditional on equal paternal age (one-sided Spearman's correlation test, *P* = 0.47; GLM *P* = 0.831) (Supplementary Fig. 6). This analysis provided no evidence that advanced maternal age causes additional postzygotic mutations to accumulate on paternally inherited chromosomes. Simulations, however, suggest that our power to detect weak postzygotic maternal age effects is limited (Supplementary Fig. 7) (*Materials and methods*).

### Nucleotide content modifies parental age and sex effects on STR DNM rate

A thorough characterization of the maternal age effect on SNV DNM rate found that the effect was enriched for specific genomic hotspots and C > G mutations (Jónsson *et al*. 2017; Gao *et al*. 2019). These attributes help suggest that the maternal age effect could be partially explained by the repair of double-strand DNA breaks. Similarly, we tested whether the STR maternal age effect was biased toward genomic hotspots or characteristic loci.

Certain mutational pathways display biases toward expansions or deletions (Alexandrov *et al*. 2020). Deletion mutations (loss of repeat unit(s)) are less common than expansion mutations (gain of repeat unit(s)) only in the maternal lineage, possibly indicating sex-specific differential mutational pathway activity (Mitra *et al*. 2021). However, we observed no significant difference in parental age effect between deletion and expansion mutations (*P* = 0.54287 and 0.212928 for *y*-intercepts, 0.89 and 0.67 for slopes, for paternal and maternal lineages, respectively) (Supplementary Fig. 8).

A set of genomic regions in humans have been classified as maternal age hotspots because they have exceptionally elevated mutation rates in the children of older mothers with a particular enrichment of C > G mutations (Jónsson *et al*. 2017). These regions appear to be hotspots for double-stranded breaks in older oocytes, leading to damage-associated mutagenesis. However, we detected no significant association between maternal age and the number of maternally derived STR DNMs (*P* = 0.99 and 0.99, Poisson GLM with identity link and Pearson's correlation test, respectively). These analyses, however, could be underpowered due to the relatively small number of STRs that both passed our filters and occurred in maternal hotspots (*n* = 432).

Kristmundsdottir *et al*. (2023) previously found that maternal and paternal ages affect the DNM rate of STRs differently as a function of their nucleotide composition. We separated STRs into two categories: STRs composed entirely of A/T nucleotides and those including any G/C nucleotides; these categories were chosen to maximize statistical power (Fig. 2a). We then modeled the effect of parental age on STR DNM rates, testing for covariance and interaction with STR nucleotide content. To account for differences in the number of loci between nucleotide content bins, we included an offset variable of the number of genotyped nonhomopolymer sites, which forces a beta coefficient of 1 (though using an offset is more straightforward to apply with a log link function, which we used in this figure's analyses) (Fig. 2a). Paternal mutation rate at GC-containing STRs was significantly higher than that of AT-only STRs, though the paternal age effect was not significantly different; in fact, a model without an interaction variable was a better fit while accounting for differences in degrees of freedom (Fig. 2b) (*P* < 2 ∗ 10^−16^ for GC content effect on intercept; delta AIC = −2). However, maternal age affected the rate of STR DNMs at AT-only STRs significantly more strongly than that at GC-containing STRs (Fig. 2c) (*P* = 0.022 for interaction between maternal age and GC content). A model without the interaction variable fit significantly worse, even while accounting for fewer degrees of freedom (delta AIC = −3). Maternal age does not significantly covary with the rate of phasing of either AT-only or GC-containing STR DNMs (*P* = 0.163 or 0.893, respectively; binomial regression).

Certain motifs can form non-B DNA conformations that affect local mutation rate; we therefore tested the maternal age effect on specific unique motifs and their predicted conformations (Guiblet *et al*. 2018; McGinty and Sunyaev 2023). We classified STR loci by their propensity to form H-DNA and Z-DNA (polypurine or polypyrimidine and purine–pyrimidine with G on one strand, respectively). Z-DNA motifs did not demonstrate a significantly different maternal age effect (*P* = 0.81, Poisson GLM with offset), although STRs likely to form Z-DNA had a significantly higher mutation rate than non-Z-DNA loci (*y*-intercept, *P* = 6.64 ∗ 10^−15^). A model testing for an interaction between H-DNA formation and maternal age demonstrated that the mutation rate at H-DNA motifs was significantly less affected by maternal age than the rate at non-H-DNA motifs, but the model's fit was inferior to a simpler model ignoring DNA shape (*P* = 0.014, Poisson GLM with offset; delta AIC = −1918). After collapsing STR motifs by periodicity and reverse complementation (i.e. grouping together loci with AC, CA, GT, and TG motifs), we estimated the maternal age effect on STRs of each common motif separately (>20 DNMs called that passed filters). The two motifs that harbored significant maternal age effects were AC and AAAT motifs (*P* < 0.05, Bonferroni correction); neither motif is self-complementary and therefore not predicted to commonly form hairpins (Guiblet *et al*. 2018).

In summary, we found that nucleotide content modifies the maternal age effect on STR DNMs. Few other *cis*-acting attributes similarly modify parental age effects; the consistency of the maternal age effect across different variables supports its validity.

### No significant effects of parental age or sex on DFE of STR DNMs

Prior research on this cohort has shown that probands diagnosed with ASD harbor more DNMs at STRs than their unaffected siblings and that these mutations are predicted to be more deleterious (Mitra *et al*. 2021). This evidence supports claims that ASD may be somewhat related to DNM burden at STRs. Following our finding that maternal age more strongly affected certain types of STRs, we sought to test whether parental age and sex affected the deleteriousness of DNMs. Selection coefficients were inferred for ∼90,000 di-, tri-, and tetranucleotide STRs using SISTR as described in (Mitra *et al*. 2021). Briefly, SISTR infers parameter *s* by fitting allele frequencies observed in the unaffected parents at each site to a model that incorporates mutation, demographic history, and selection. This parameter refers to the increase in the selection coefficient (i.e. decrease in reproductive fitness) associated with each additional repeat unit away in length from the major allele, assumed here to be the most fit. As in Mitra *et al*., we excluded sites for which the posterior distribution of *s* was greater than 0.3, indicating poor model fit; this left ∼63,000 sites. Notably, our additional filters accounting for possible allelic dropout removed 38.1% (20,419 of 53,602) STR DNMs for which a selection coefficient could be inferred.

To initially test for parental age effects on the DFE of STR DNMs, we aggregated the allele-specific selection coefficients of paternally and maternally derived mutations across all children born to parents below and above the median age for each sex, respectively. We found no significant differences in the DFE (two-sided Kolmogorov–Smirnov test, *P* = 0.7487 and 0.292, respectively). We also modeled the effects of parental age and sex on DFE with Poisson models regressing the number of paternal and maternal STR DNMs at constrained or neutral sites as a function of parental age. Neutral loci were those with an *s* = 0; constrained loci had an *s* in the upper 90th percentile of all genotyped loci with *s* values. In these Poisson models, we tested for linear interaction between constraint and parental age while accounting for differences in the number of loci with an offset variable. The interaction terms were not significant for either parental sex, indicating no significant effect of parental age or sex on DFE (*P* = 0.459 and 0.21591). Furthermore, in contrast to Mitra *et al*., we did not observe any significant differences in the DFE between STR DNMs found in probands and unaffected siblings in the database (two-sided Kolmogorov–Smirnov test on selection coefficients summed across all children by diagnosis, *P* = 0.515). This difference may simply reflect the lower statistical power associated with our strict filtering methods, as described above.

## Discussion

Our analyses of the parental age effects on STR mutagenesis support the hypothesis that STR mutagenesis is more replication dependent than SNV mutagenesis but do not support the dogma that replication slippage during S phase is the sole cause of STR mutations. The fraction of phased mutations deriving from the paternal lineage increases with paternal age, supporting a hypothesis that the ratio of paternal to maternal mutation rates is lower prepuberty than postpuberty. This is ostensibly a result of the continued replication of the paternal germline after puberty. Nevertheless, the significant maternal age effect cannot be a result of polymerase slippage during S-phase replication, as the maternal germline does not replicate after birth (Drost and Lee 1995). This observation is reminiscent of findings of STR expansions in postmitotic cells and may provide broader evidence that damage-associated STR mutagenesis is observed across many cell types (Gonitel *et al*. 2008).

The stronger maternal age association with deletion DNMs at AT-only repeats may provide a clue as to the source of these mutations, though our current understanding of STR mutational signatures is not advanced enough to decode it. A number of indel mutational signatures in COSMIC, a database of signatures deconvoluted from mutation spectra identified in tumors, have proposed etiologies such as TOP1 transcription-associated mutagenesis (Alexandrov *et al*. 2020; Reijns *et al*. 2022). However, no specific indel signature matches well with our observed maternal age effect, enriched at AT repeats but without bias toward expansions and deletions. Future work may further integrate this mutational data with somatic mutational data, particularly from postmitotic cells, to help elucidate possible pathways.

## Supplementary Material

iyae013_Supplementary_Data

## Data Availability

Code to generate figures and run statistical tests is available on GitHub at https://github.com/goldmich/str_dnm. VCFs and STR DNMs published in Mitra *et al*. (2021) are available through SFARI to approved researchers at SFARI Base with base accession code: SFARI_SSC_WGS_2b. Phenotype and sequencing data for the SSC are also available through SFARI Base (accession numbers SFARI_SSC_WGS_p, SFARI_SSC_WGS_1, and SFARI_SSC_WGS_2). HiFi and ONT sequencing data for 2 quads have been deposited to SFARI base (accession number SFARI_DS714840). liftOver and associated files for the comparison with Sun *et al*. (2012) were downloaded from the UCSC table browser (https://genome.ucsc.edu/cgi-bin/hgTables). Supplemental material available at GENETICS online.
