# Supplementary material for: Effects of parental age and polymer composition on short tandem repeat de novo mutation rates
Source: Genetics. 2024 Jan 31;226(4):iyae013. doi: 10.1093/genetics/iyae013 (PMC10990422; doi:10.1093/genetics/iyae013)
Supplement: iyae013_Supplementary_Data [file iyae013_supplementary_data.zip › Supplemental_Figure_Legends_GENETICS-2023-306753.docx]

Figure S1: Inheritance bias potentially supports allelic dropout. (A) We observed that, prior to filtering, DNMs at loci where one parent was heterozygous and the other was homozygous nearly always phased to the homozygous parent. Here we have plotted the fraction of mutations per child at those types of sites that phase to the homozygous parent. (B) Once we filter out DNMs where ≥ 1 read in the parent contains the putative *de novo* allele, we observe a reduction in the skew towards inheritance from the homozygous parent. The behavior following this filtering supports allelic dropout as a possible erroneous source of DNMs.

Figure S2: Variance in filtration sensitivity between genotypes at the same locus (chr22: 27079676). We filter out any STR DNM when >0 reads across either parent support the putative *de novo* allele. This filtering strategy is strict; we therefore explored the effect on filter sensitivity of a allowing a higher threshold of parental reads supporting the de novo allele. Putative *de novo* alleles were identified in children of two different families at the same locus; parental and child genotypes in the first family were [11,11], [11,11], and [11,10], respectively; the second family were [11,11], [11,13], and [11,12], respectively. To estimate sensitivity of our filtration strategy, we identified a set of “positive” couples with identical genotypes to the targeted family but no putative *de novo* allele in their children; we believe that allelic dropout is unlikely to have occurred when genotyping these positive couples. To assess how frequently we would erroneously flag these couples’ genotypes for allelic dropout and explore whether to allow a higher threshold of reads mapping to a putative *de novo* allele. Plotted in the histograms are the distribution of the number of reads, summed between both parents, that support the putative *de novo* allele in the positive couples. Filter sensitivity is higher for couples with [11,11] and [11,13] than for couples with [11, 11] and [11,11].

Figure S3: Homopolymers do not harbor parental age effects. We plotted the number of STR DNMs for each trio in the SSC as a function of their parental lineage (paternal vs. maternal) and length of repeat unit (homopolymer vs. nonhomopolymer). Poisson GLMs with identity link functions were fit using ggplot2. Homopolymers have no significant association with either paternal or maternal age. Y-axis jitter was added for readability.

Figure S4: No detectable maternal age effect on loci identified in Kong et al., 2002. Sun et al., 2012 reported no significant maternal age effect on STR DNM. To replicate their analyses in the SSC, we subset our variants to those that overlapped loci from Kong et al., 2002 (N = 2040). We then regressed the maternal mutation rate against maternal age and found no significant association (*P* = 0.165, GLM). The figure above shows the distribution of maternal mutation rates as a function of maternal age deciles; the GLM line was fit with ggplot2.

Figure S5: Positive association of paternal age with paternal fraction of mutations. We plotted alpha, the fraction of paternally phased mutations of all phased mutations per trio in the SSC. Quasibinomial regression line with identity link fit with ggplot2. Y-axis jitter was added for readability.

Figure S6: No evidence for postzygotic effect of maternal age on STR DNM rate. Nonoverlapping pairs of children were grouped together by identical (+/- 6 months) paternal ages. Each pair is represented a single time in the scatterplot above, where the difference in the number of paternally derived STR DNMs between the kids is plotted as a function of the difference in maternal age (older – younger). GLM regression line fit with ggplot2, though the slope is not significantly different from zero. X-axis jitter was added for readability.

Figure S7: Limited power to detect maternal age effect on postzygotic STR DNMs. The postzygotic fraction represents the fraction of STR DNMs associated with the maternal age effect that occur after zygote formation and are therefore randomly distributed between the paternally and maternally inherited chromosomes. We simulated paternally derived STR DNMs with a variety of postzygotic fractions for each child in the SSC. We then determined the correlation between these (simulated) paternally derived STR DNMs and the difference in maternal age between pairs of children born to fathers of the same ages, as in Figure S6. Plotted are the fraction of *P* values from a one-sided Spearman’s rank correlation test that are significant to *P* < 0.05 from 500 simulations at each postzygotic fraction.

Figure S8: No parental age effects specific to mutation direction. Each trio in the SSC is represented twice on each scatterplot, where the points represent the number of paternal or maternally derived deletion or expansion STR DNMs. Poisson GLMs with an identity link function fit with ggplot2. The slope coefficient of paternal and maternal age is not significantly different between expansions and deletions. Vertical jitter added for readability.
