# Supplementary figures and images for: Effects of parental age and polymer composition on short tandem repeat de novo mutation rates
Source: Genetics. 2024 Jan 31;226(4):iyae013. doi: 10.1093/genetics/iyae013 (PMC10990422; doi:10.1093/genetics/iyae013)

**A**

count

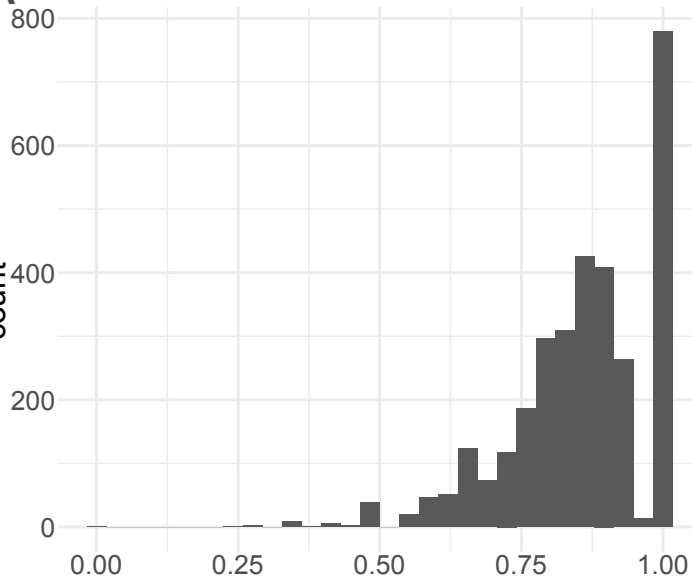**B**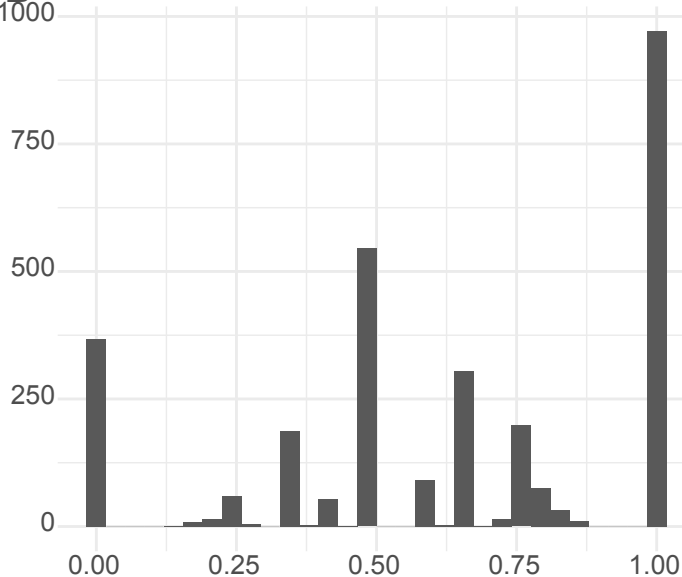

Supplement: iyae013_Supplementary_Data [file iyae013_supplementary_data.zip › Figure_S1_GENETICS-2023-306753.pdf]

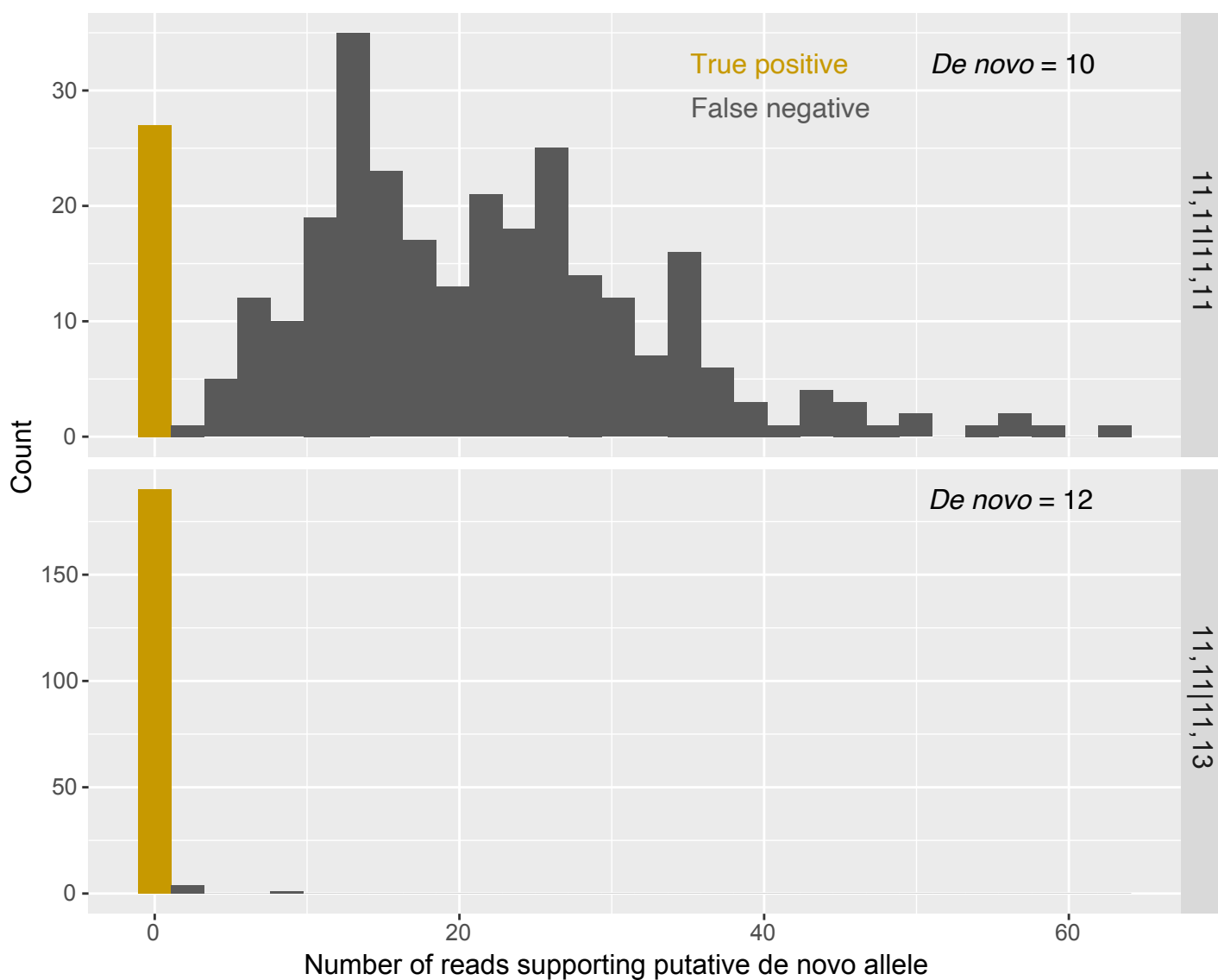

Supplement: iyae013_Supplementary_Data [file iyae013_supplementary_data.zip › Figure_S2_GENETICS-2023-306753.pdf]

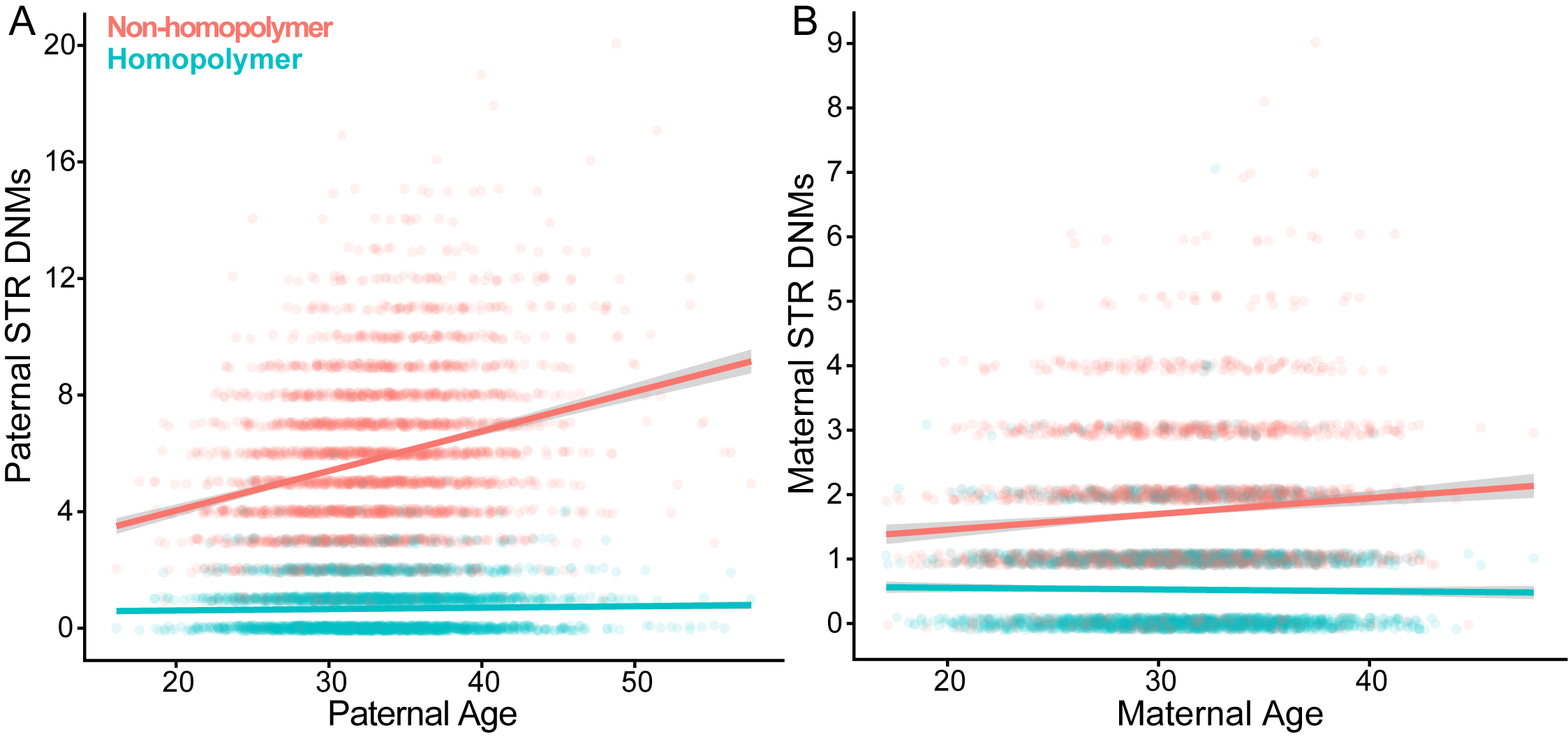

Supplement: iyae013_Supplementary_Data [file iyae013_supplementary_data.zip › Figure_S3_GENETICS-2023-306753.tif]

STR DNM rate

paternal  
maternal

$4e-04$

$2e-04$

$0e+00$

25

Median age (yr)

30

35

40

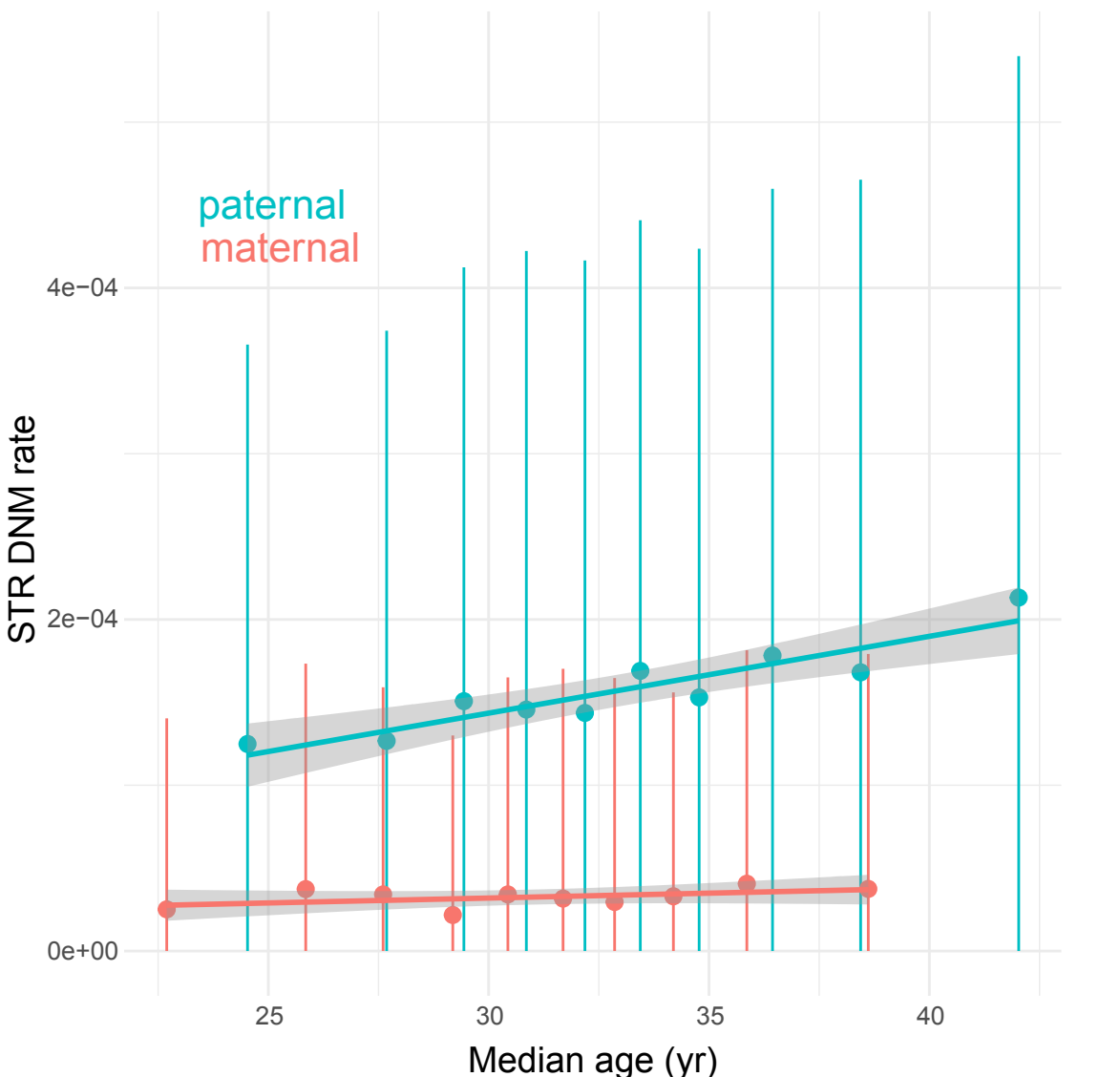

Supplement: iyae013_Supplementary_Data [file iyae013_supplementary_data.zip › Figure_S4_GENETICS-2023-306753.pdf]

Paternal fraction of STR DNMs

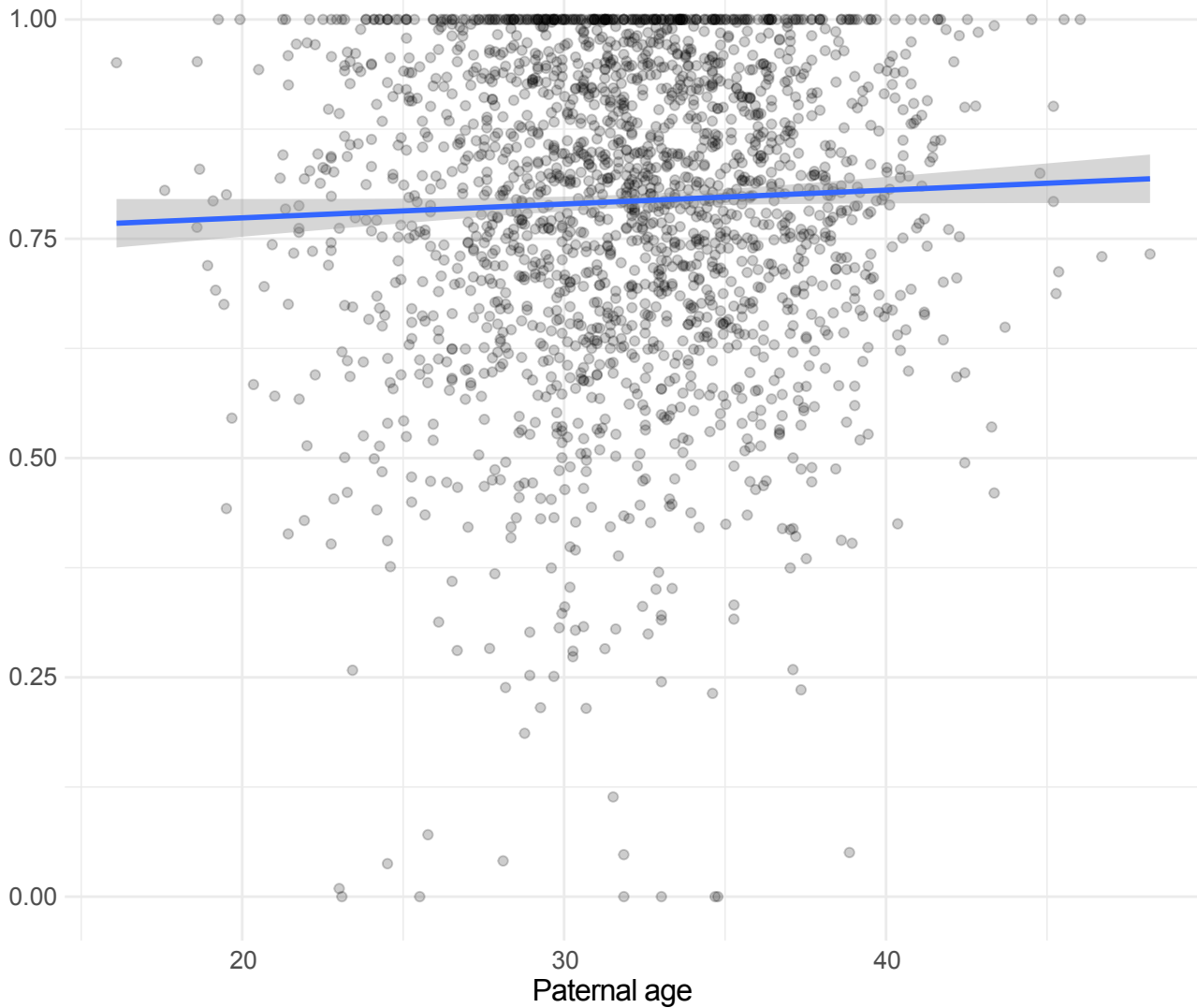

Supplement: iyae013_Supplementary_Data [file iyae013_supplementary_data.zip › Figure_S5_GENETICS-2023-306753.pdf]

Difference in paternal STR DNMs

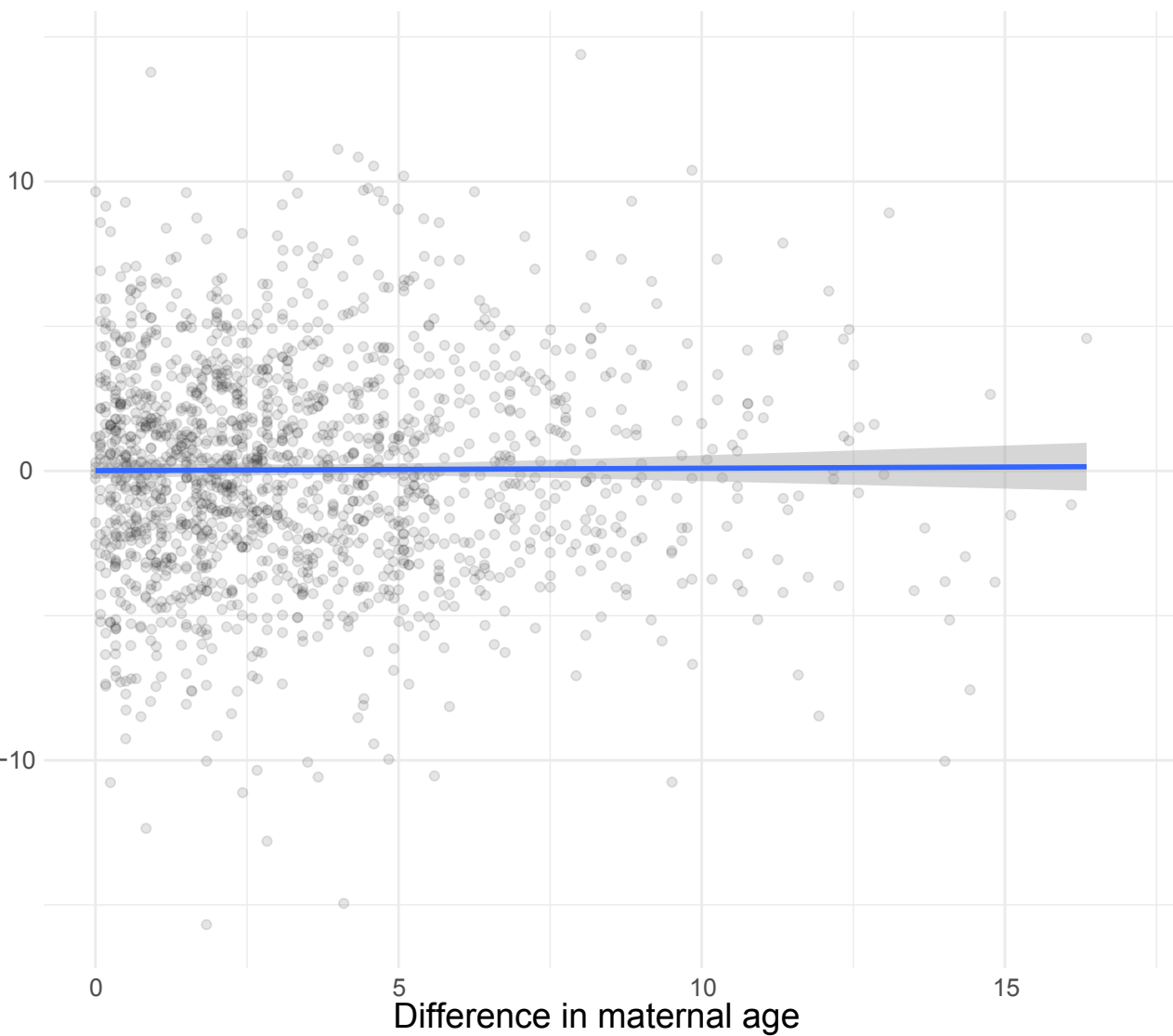

Supplement: iyae013_Supplementary_Data [file iyae013_supplementary_data.zip › Figure_S6_GENETICS-2023-306753.pdf]

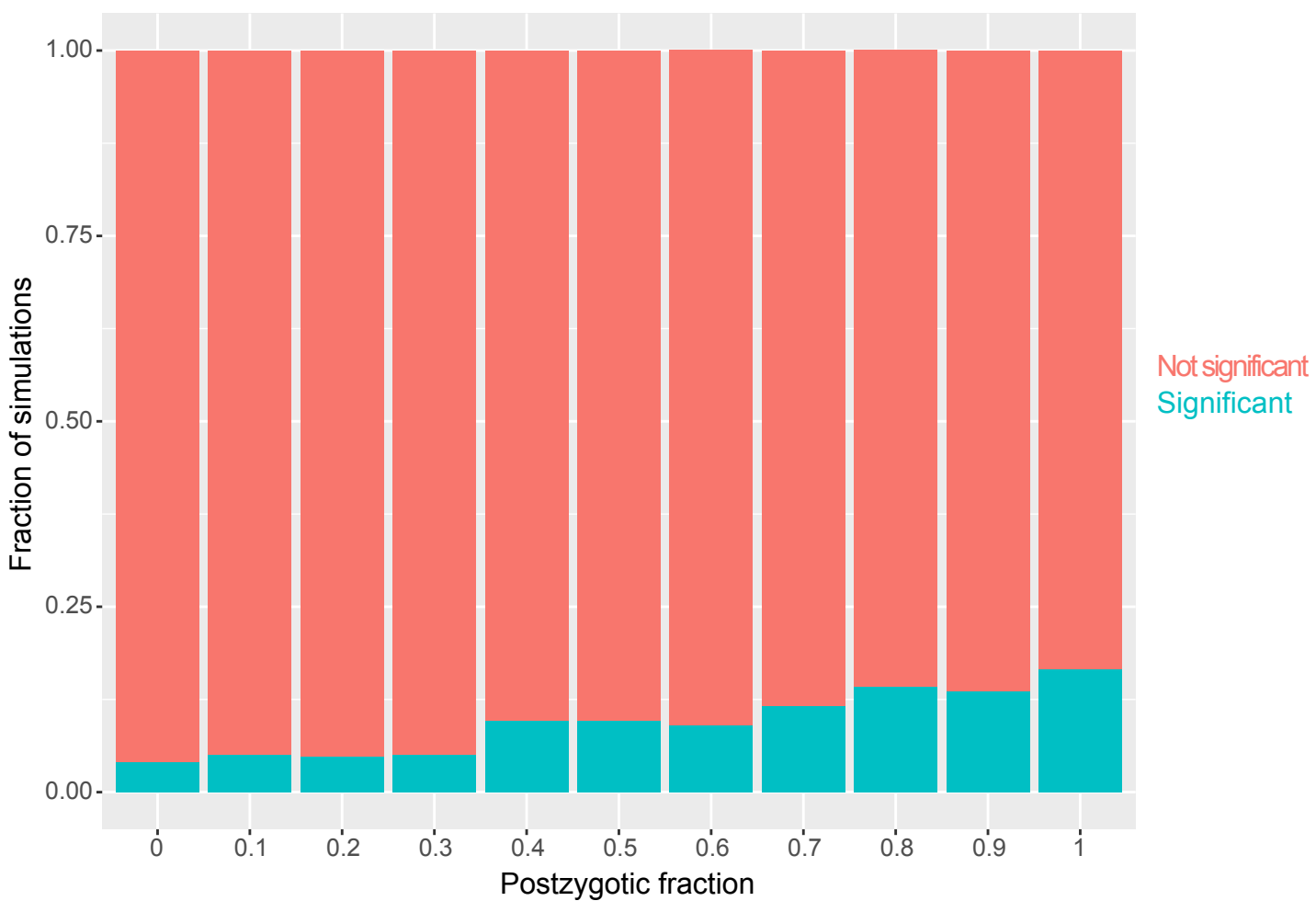

Supplement: iyae013_Supplementary_Data [file iyae013_supplementary_data.zip › Figure_S7_GENETICS-2023-306753.pdf]

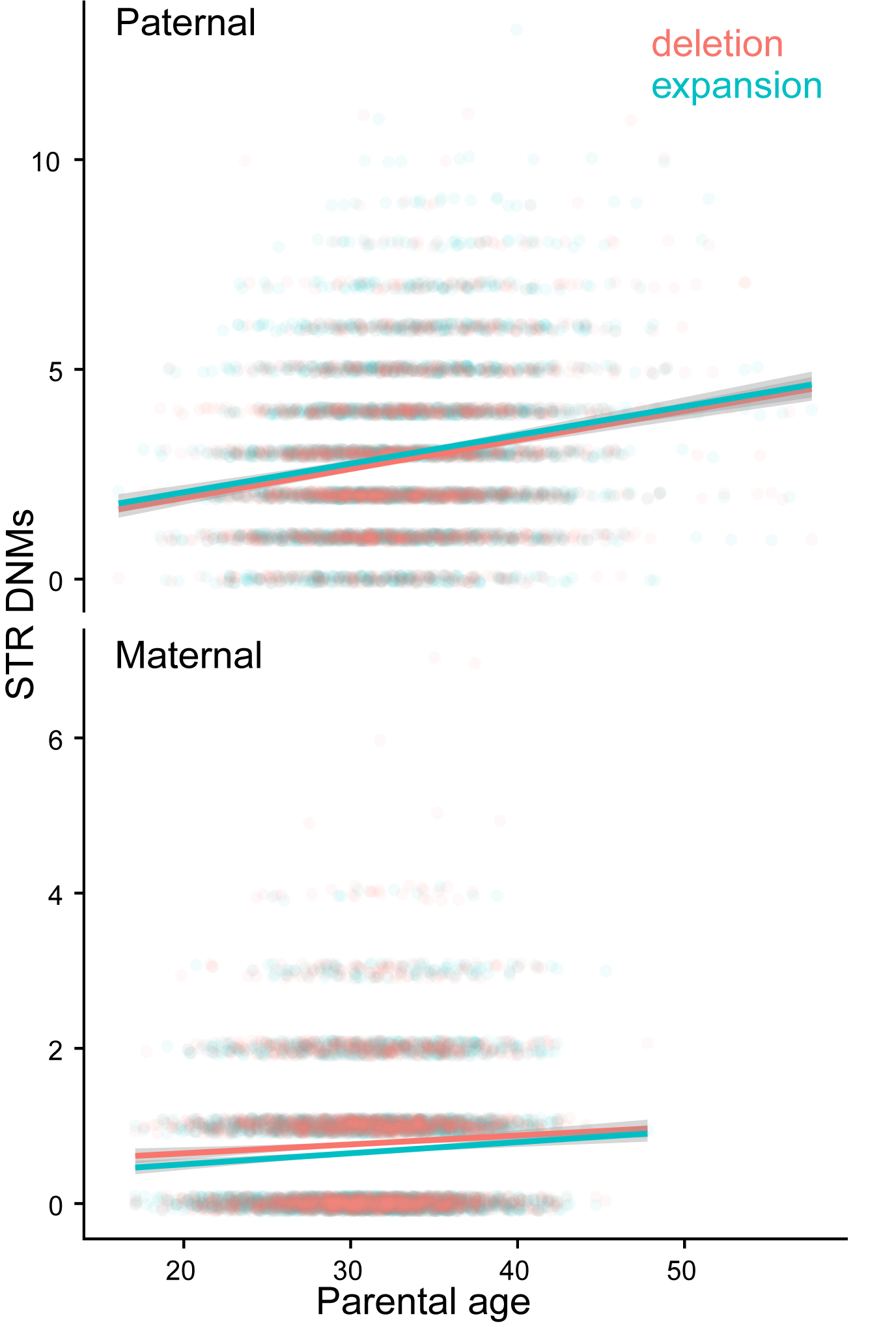

Supplement: iyae013_Supplementary_Data [file iyae013_supplementary_data.zip › Figure_S8_GENETICS-2023-306753.tif]
